# Generation and analysis of transcriptomic resources for a model system on the rise: the sea anemone *Aiptasia pallida *and its dinoflagellate endosymbiont

**DOI:** 10.1186/1471-2164-10-258

**Published:** 2009-06-05

**Authors:** Shinichi Sunagawa, Emily C Wilson, Michael Thaler, Marc L Smith, Carlo Caruso, John R Pringle, Virginia M Weis, Mónica Medina, Jodi A Schwarz

**Affiliations:** 1 School of Natural Sciences, University of California, Merced, CA 95344, USA; 2 Biology Department, Vassar College, Poughkeepsie, NY 12604, USA; 3 Computer Science Department, Vassar College, Poughkeepsie, NY 12604, USA; 4 Department of Genetics, Stanford University School of Medicine, Stanford, CA 94305, USA; 5 Department of Zoology, Oregon State University, Corvallis, OR 97331, USA

## Abstract

**Background:**

The most diverse marine ecosystems, coral reefs, depend upon a functional symbiosis between cnidarian hosts and unicellular dinoflagellate algae. The molecular mechanisms underlying the establishment, maintenance, and breakdown of the symbiotic partnership are, however, not well understood. Efforts to dissect these questions have been slow, as corals are notoriously difficult to work with. In order to expedite this field of research, we generated and analyzed a collection of expressed sequence tags (ESTs) from the sea anemone *Aiptasia pallida *and its dinoflagellate symbiont (*Symbiodinium *sp.), a system that is gaining popularity as a model to study cellular, molecular, and genomic questions related to cnidarian-dinoflagellate symbioses.

**Results:**

A set of 4,925 unique sequences (UniSeqs) comprising 1,427 clusters of 2 or more ESTs (contigs) and 3,498 unclustered ESTs (singletons) was generated by analyzing 10,285 high-quality ESTs from a mixed host/symbiont cDNA library. Using a BLAST-based approach to predict which unique sequences derived from the host versus symbiont genomes, we found that the contribution of the symbiont genome to the transcriptome was surprisingly small (1.6–6.4%). This may reflect low levels of gene expression in the symbionts, low coverage of alveolate genes in the sequence databases, a small number of symbiont cells relative to the total cellular content of the anemones, or failure to adequately lyse symbiont cells. Furthermore, we were able to identify groups of genes that are known or likely to play a role in cnidarian-dinoflagellate symbioses, including oxidative stress pathways that emerged as a prominent biological feature of this transcriptome. All ESTs and UniSeqs along with annotation results and other tools have been made accessible through the implementation of a publicly accessible database named AiptasiaBase.

**Conclusion:**

We have established the first large-scale transcriptomic resource for *Aiptasia pallida *and its dinoflagellate symbiont. These data provide researchers with tools to study questions related to cnidarian-dinoflagellate symbioses on a molecular, cellular, and genomic level. This groundwork represents a crucial step towards the establishment of a tractable model system that can be utilized to better understand cnidarian-dinoflagellate symbioses. With the advent of next-generation sequencing methods, the transcriptomic inventory of *A. pallida *and its symbiont, and thus the extent of AiptasiaBase, should expand dramatically in the near future.

## Background

Many biological systems rely on symbiotic interactions between different organisms. One of the most dramatic examples is the coral reef ecosystem, which has at its heart a mutualistic partnership between corals and endosymbiotic, dinoflagellate algae. The dinoflagellates are classified in a single genus, *Symbiodinium*, but molecular methods have revealed a high genetic diversity in this genus [1,2]. The onset of these symbioses has been shown to display flexibility, but a range of specificity, i.e. from highly flexible to highly specific, is apparent during its maintenance [3-8]. This process is likely to involve early recognition mechanisms [9,10] and an evasion of the hosts' digestive and immune systems [11], as well as adaptations to diverse ecological niches [12,13] and physiological acclimation [14,15]. There have also been controversial discussions of whether *Symbiodinium *populations may shift toward more heat-tolerant types as a consequence of thermal stress ("bleaching") in order to adapt to environmental changes [16-18] such as increasing seawater temperatures. In light of global climate change, this subject, i.e. cnidarian bleaching, has received much attention as devastating mass bleaching events have increased both in frequency and geographic extent [19]. Nonetheless, our knowledge of the underlying cellular and molecular mechanisms that facilitate the recognition between the partners, and determine the specificity, dynamics, and collapse of cnidarian-dinoflagellate symbioses, is limited.

The cellular and molecular interactions between host and symbiont cells are important targets for genetic and genomic dissection, but corals are notoriously difficult to work with. For example, corals form large, slow-growing colonies that are difficult and costly to maintain in the laboratory, and their handling for microscopy and amenability to other cell biological, biochemical, and genetic methods is complicated by the calcareous skeleton precipitated by reef-building corals. What is needed to make rapid advances in this field is a model system that possesses the key characteristics of coral symbiosis, but allows more facile laboratory investigation (for a detailed review see [20]). The sea anemone *Aiptasia *represents a good candidate system [20], as it possesses the same mutualistic relationship with *Symbiodinium *spp., but lacks the calcareous skeleton that hinders cellular-level work. It is widely distributed, and found in shallow tropical marine environments worldwide. Sequence characterized amplified region (SCAR) data indicate that the vast majority of *Aiptasia *worldwide (encompassing two described species, *A. pallida *and *A. pulchella*), appear to be genetically homogeneous (Santos Lab at Auburn University, pers. comm.). The one exception is a closely related, but genetically distinct, lineage potentially restricted to the Florida Keys. Data from the Santos Lab also indicate that natural populations of *Aiptasia *from the Florida Keys preferentially host *Symbiodinium *spp. comprised of only clade A or both clades A and B, whereas those from the remaining global range host clade B exclusively. Typically considered a pest organism by seawater aquarists, *Aiptasia *is hardy and proliferates rapidly by asexual reproduction. Individual polyps can be maintained in a symbiotic or aposymbiotic state (i.e., with and without symbionts, respectively), experimentally re-infected with a variety of *Symbiodinium *strains [21,22], and cultured at low cost [23]. In fact, numerous studies have addressed symbiosis-related questions using *A. pallida *and its sister species *A. pulchella *by applying multiple tools ranging from microscopy to RNA-interference methods [24-29]. The generation of genomic resources for *Aiptasia *would therefore greatly advance research addressing the understanding of symbioses at a molecular, cell-biological, and genomic level.

As a cost-effective alternative to sequencing the genome of an organism, the generation and analysis of expressed-sequence-tag (EST) libraries provides an efficient method for discovering novel genes, estimating gene content, and approximating levels of gene expression. Once established, these resources can be utilized for comparative genomics studies or the construction of gene expression microarrays [30]. Among cnidarians, the extensive genomic resources now available for the non-symbiotic sea anemone *Nematostella vectensis *have opened new perspectives on the study of basal metazoans [31], and several EST resources have been generated for symbiotic cnidarians (predominantly corals) and *Symbiodinium *spp. [32-34]. However, to date, only one small-scale project has generated ESTs (N = 870) for the symbiotic anemone *Aiptasia pulchella *[35].

In this study, we report the generation and analysis of 10,285 high-quality ESTs from a *Symbiodinium *clade A-hosting clonal population of *Aiptasia pallida *that was likely derived from an individual originating from the Florida Keys lineage, which were processed through a software pipeline [36] resulting in a user-friendly, queryable, web-accessible database named AiptasiaBase. A BLASTx-based approach was used to estimate the relative contributions of each partner to the mixed cDNA library, and we were able to identify numerous genes involved in key processes of cnidarian-dinoflagellate symbioses.

## Results and Discussion

### EST library construction and assembly

A total of 6,448 cDNA clones were bi-directionally sequenced, resulting in 12,896 raw chromatograms, which served as input for the processing pipeline. After base calling by phred [37], Lucy [38] discarded 2,556 low-quality sequences, short or insert-less sequences, and vector or polyA-only sequences. An additional 55 sequences were removed by seqclean [39], leaving 10,285 high-quality ESTs (from 5,450 cDNA clones) for further processing (success rate ~80%). Assembly of these ESTs by cap3 [40] resulted in the generation of 1,427 contigs, which ranged from 112 to 3,440 bp in length and contained 2 – 259 ESTs (mean: 4.8). Together with the remaining 3,498 singletons, a total of 4,925 unique sequences (UniSeqs) were generated. Because of the possibility that two (or more) UniSeqs originated from the same transcript, we also estimated the number of unique genes (unigenes) in our dataset by assembling only the reverse reads of the directionally cloned cDNAs. The resulting estimate of 2,564 unigenes compared to the 4,925 UniSeqs is likely to reflect the large average size (1.95 kb) of inserts in the cDNA library; thus, in many cases, UniSeqs represent the 3' and 5' ends of genes for which the central parts were not captured due to Sanger-sequencing length limitations (600–800 bp). In addition, different splice variants or alleles of the same gene may have contributed to the excess of UniSeqs over unigenes. Detailed pre-assembly statistics are summarized in Additional file 1: Quality control and assembly statistics.

Previously, a small-scale EST project was conducted in order to compare the abundance of transcripts between symbiotic and aposymbiotic *Aiptasia pulchella *polyps [35]. The present study included bi-directional sequencing, and the total number of ESTs is more than 14 times larger than in the earlier study. Therefore, the availability of almost 5,000 UniSeqs for about 2,500 unigenes represents a rich transcriptomic resource, previously unavailable at this scale, for a symbiotic anemone.

### Annotation of unique sequences and implementation of AiptasiaBase

All UniSeqs were assigned putative identities based on BLASTx hits (E-value cutoff: 1e-5) to the UniProt Knowledgebase databases SwissProt and TrEMBL [41]. About 37% and ~63% of the UniSeqs found hits in SwissProt and TrEMBL, respectively, leaving ~36% of the UniSeqs without similarities to known proteins. Because the TrEMBL database contains protein sequences based on conceptual translations of all nucleotide sequence entries in EMBL/GenBank/DDBJ, we chose to annotate the UniSeqs according to the curated SwissProt entries. Assignments of gene ontologies (GO) could be made for about one third of UniSeqs in each of the GO categories: molecular function, biological process, and cellular component. Because our cDNA library represents the symbiotic, adult life-history stage of *A. pallida*, the GO resource generated in this study sets the stage for statistical assessments of over- or under-representation of specific GO-categories in libraries obtained from anemones under different conditions such as life-history stages, symbiotic state (symbiotic vs. aposymbiotic), or environmental conditions (temperature, salinity, nutrients, etc.). In addition to BLAST and GO annotations, all UniSeqs were screened for single-nucleotide polymorphisms (SNPs) and simple-sequence repeats (SSRs), providing resources for the investigation of gene polymorphisms between individuals and/or populations. The prediction of open reading frames within UniSeqs also provided the basis for domain annotations at the protein level. About 25% of UniSeqs matched a protein domain entry in the Pfam database [42].

One of the primary challenges of sequencing ESTs from a mixed transcriptome originating from two or more partners is to assign sequences to the proper genome of origin. Taking a bioinformatic approach to this problem, we constructed taxon-specific databases representing either "Cnidaria-only" or "Alveolata-only" (i.e., dinoflagellates and their relatives) sequences from GenBank, and then performed BLASTx-searches against those databases as well as the complete non-redundant database (see Methods). We then employed a best-BLASTx-hit (BBH) approach (Additional file 2: Flow diagram illustrating BBH approach) to estimate the numbers of ESTs that originated from *A. pallida *and *Symbiodinium *spp., respectively, at various levels of confidence (Table 1). Irrespective of the confidence level, about one quarter of ESTs had no BLASTx-hit (E-value cutoff 1e-5). At the different levels of confidence, 56 – 70% and 1.7 – 6.4% were predicted to originate from the anemone and the *Symbiodinium *genomes, respectively (Additional file 3: Detailed EST (N = 10,285) distribution and assignment). The relatively small fraction of *Symbiodinium *ESTs could be expected given that *Symbiodinum *spp. are spatially restricted to the endodermal tissue layer of the host and that no special effort was made to disrupt the algal cell walls during the preparation of the RNA (see Methods). Furthermore, the number of UniSeqs without a significant BLASTx hit may be higher for *Symbiodinium *transcripts. However, the uncertainty about the origin of non-annotated sequences represents a current limitation to our approach. Ongoing and future genome-sequencing projects for symbiotic cnidarians and their dinoflagellate endosymbionts should soon become available and help to uncover the origins of sequences without currently known homologs in other organisms. This will provide an interesting opportunity to revisit our data set to look further at these perhaps taxonomically restricted genes.

**Table 1:** Predicted genome-of-origin for contigs and singletons of the holobiont

**Type of UniSeq**	**# of UniSeqs**	**Predicted genome of origin**	**Prediction assignment**	**Confidence**	**# of ESTs (%)**
Contig	775	*Aiptasia*	BBH = Cnidaria	high	4029 (39)
Contig	32	*Symbiodinium*	BBH = Alveolata	high	88 (0.86)
Contig	144	*Aiptasia*	E-value(Cnidaria) < E-value(Alveolata)	medium	540 (5.3)
Contig	63	*Symbiodinium*	E-value(Alveolata) < E-value(Cnidaria)	medium	228 (2.2)
Contig	14	*Aiptasia*	sim%(Cnidaria) > sim%(Alveolata)	low	576 (5.6)
Contig	7	*Symbiodinium*	sim%(Alveolata) > sim%(Cnidaria)	low	27 (0.26)
Contig	2	n/a	no assignment	-	5 (0.049)
Contig	390	n/a	no hit	-	1294 (12)

*Sum*	*1427*				*6787 (66%)*

Singleton	1731	*Aiptasia*	BBH = Cnidaria	high	1731 (16)
Singleton	91	*Symbiodinium*	BBH = Alveolata	high	91 (0.88)
Singleton	307	*Aiptasia*	E-value(Cnidaria) < E-value(Alveolata)	medium	307 (2.9)
Singleton	175	*Symbiodinium*	E-value(Alveolata) < E-value(Cnidaria)	medium	175 (1.7)
Singleton	42	*Aiptasia*	sim%(Cnidaria) > sim%(Alveolata)	low	42 (0.41)
Singleton	49	*Symbiodinium*	sim%(Alveolata) > sim%(Cnidaria)	low	49 (0.48)
Singleton	1103	n/a	no hit	-	1103 (10)

*Sum*	*3498*				*3498 (34%)*

Details can be found in Additional file 2: Flow diagram illustrating BBH appraoch and Additional File 3: Detailed EST (N = 10,285) distribution and assignment.

Using an EST-processing software (EST2uni) [36], we stored all ESTs, UniSeqs, and annotations in a queryable database named AiptasiaBase (database = AiptasiaBase_v1), which is accessible through the URL: . In addition to the results generated by the software, we have included the annotation of UniSeqs according to KEGG, which provides a convenient way to explore pathway components that were identified in this study.

### Analysis of the most highly abundant transcripts

We identified the contigs containing the greatest numbers of ESTs, which we used as a proxy for the most abundant transcripts. Although the numbers of ESTs in contigs that are predicted to originate from *Symbiodinium *were too low to be analyzed (data not shown), many of the most abundant host-derived transcripts represented genes that are involved in the processes of protein biosynthesis, extracellular-matrix formation, and oxidative-stress response (Table 2).

**Table 2:** Most highly expressed genes predicted to originate from the host genome^a^

**Contigs**	**# of reads (%)**	**Annotation**	**Accession SwissProt**	**Species**	**E-value**
Contig777	259 (2.5)	Elongation factor 2	P05197	*Rattus norvegicus*	0
Contig642^b^	203 (2.0)	Elongation factor 1-alpha	P29520	*Bombyx mori*	0
Contig643^c^	181 (1.8)	Polyadenylate-binding protein 1	P11940	*Homo sapiens*	1e-177
Contig785^c^	178 (1.7)	Polyadenylate-binding protein 4	Q13310	*Homo sapiens*	8e-30
Contig248	113 (1.1)	Collagen alpha-2(I) chain	Q01149	*Mus musculus*	6e-41
Contig824^d^	110 (1.1)	Apolipophorins	Q9U943	*Locusta migratoria*	2e-33
Contig174^e^	83 (0.8)	no_hits_found	-	-	-
Contig514	79 (0.8)	Catalase	P04040	*Homo sapiens*	0
Contig1015^b^	55 (0.5)	Elongation factor 1-alpha	P29520	*Bombyx mori*	1e-125
Contig950	51 (0.5)	Collagen alpha-1(II) chain	Q91717	*Xenopus laevis*	2e-50
Contig262	50 (0.5)	no_hits_found	-	-	-
Contig6	44 (0.4)	no_hits_found	-	-	-
Contig651	43 (0.4)	Adenosylhomocysteinase	P27604	*Caenorhabditis elegans*	0
Contig1051^f^	42 (0.4)	CUB and zona pellucida-like domain-containing protein 1	Q86UP6	*Homo sapiens*	3e-23
Contig425	41 (0.4)	Peroxidasin homolog	Q92626	*Homo sapiens*	4e-88

^a ^BLASTx (E-value cutoff: 1e-5) search in SwissProt (as posted on 08/01/2008). Contig numbering is according to AiptasiaBase_v1.

^b ^Contigs 642 and 1015 are likely to originate from the same transcript.

^c ^Contigs 643 and 785 are likely to originate from the same transcript.

^d ^Best BLAST search hit (with annotation) in nr: von Willebrand factor-like protein (*Anthopleura elegantissima*) [GenBank:DQ309533].

^e ^Sequencing error artifact.

^f ^Best BLAST search hit (with annotation) in nr: mesoglein variant 1 (*Aurelia aurita*) [GenBank:EF093532].

Kuo *et al*. (2004) reported that the most highly expressed gene in symbiotic *A. pulchella *was ferritin (11.7%), whereas we found only 4 ESTs (0.04%) that represented this gene. Although differences in the preparations of the cDNA libraries (e.g. insert size-selection) and sequencing depths (474 vs. 10,285 ESTs) pose an obstacle for a direct comparison, the discrepancy in the numbers of ferritin transcripts appears to be noteworthy. In a recent study that investigated the effect of increased temperature and UV levels on the symbiotic anemone *Anthopleura elegantissima*, Richier et al. (2008) observed a more than 17-fold up-regulation of ferritin expression upon thermal stress, but not UV stress [43]. Given this observation, it seems possible that the anemones in the study by Kuo *et al*. (2004) were under elevated thermal stress at the time of sampling, which, taken together with the methodological differences mentioned above, makes any further comparative analyses unfeasible. This result has important implications, i.e., how culturing conditions of organisms as well as methodological differences between studies may have an impact on the transcriptome, and by extension, the interpretation of gene expression analyses.

The highly abundant sequences with the highest uncertainties for correct annotation (highest E-values), apolipophorin and the CUB and zona pellucida-like domain-containing protein 1, were further scrutinized by similarity searches in additional databases. These searches revealed that the best hit for CUB and zona pellucida-like domain-containing protein 1 in the GenBank non-redundant database (nr) was mesoglein, a protein that is proposed to be a structural element of the extracellular matrix of the mesoglea in the jellyfish *Aurelia aurita *[44]. The sequence annotated as apolipophorin contains a von Willebrand factor type-D domain, and was reported to be involved in forming lipoprotein particles that bind lipoproteins and lipids [45]. Two other highly abundant sequences had no homologs among previously characterized proteins, suggesting that they are novel, and a third contig with no BLASTx-hit was identified as an artifact due to misassembled sequences. These results illustrate some of the caveats to automated sequence assembly and annotation and highlight the necessity for corroboration after automated sequence processing when focusing on single genes or groups of genes of interest.

### Candidate genes with potential relevance to cnidarian-dinoflagellate symbioses

**Figure 1:**
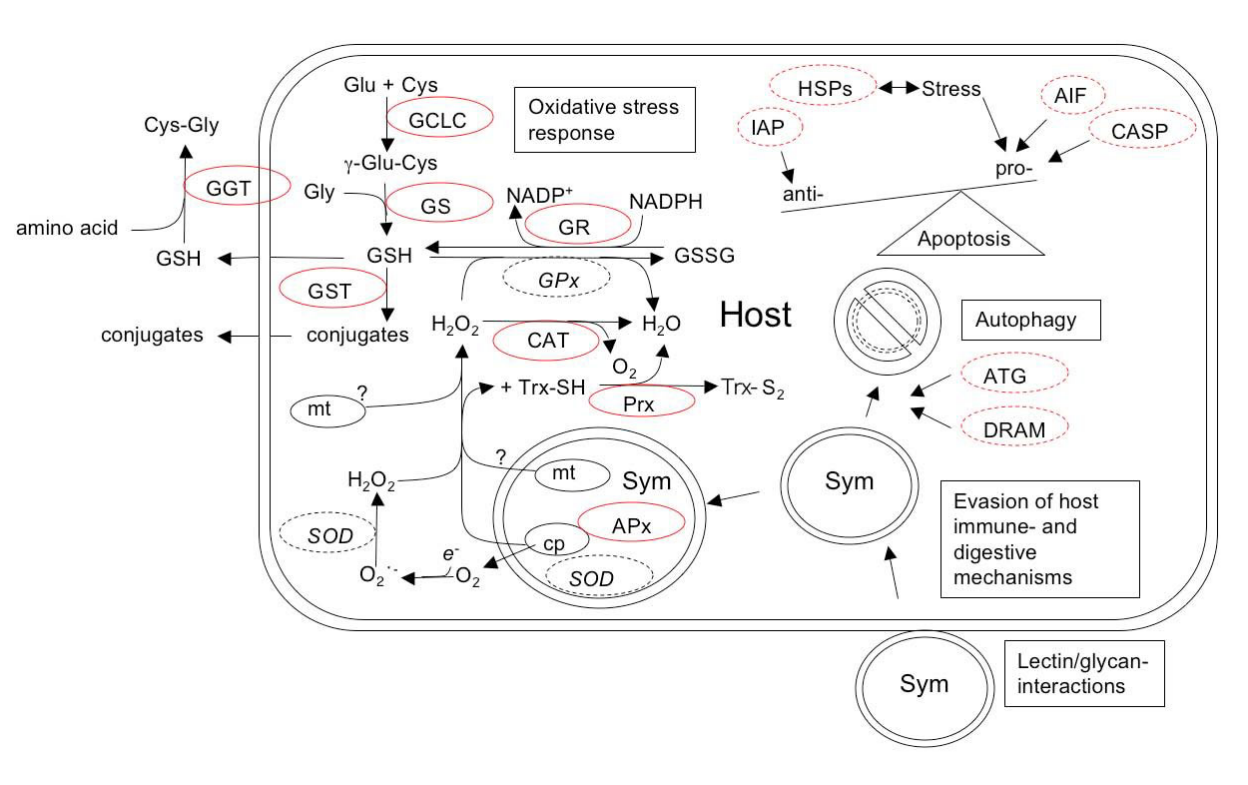
**Illustration of genes and pathways known or likely to be involved in cnidarian-dinoflagellate symbioses**. Genes that were identified or missing in the EST library are highlighted by solid red lines or dashed black lines, respectively. Pathways or cellular processes that were partially represented are highlighted by dashed red lines. APx – ascorbate peroxidase, ATG – autophagy-related protein, AIF – apoptosis-inducing factor, CASP – caspases, CAT – catalase, DRAM – damage-regulated autophagy modulator, GCLC – glutamate-cysteine ligase catalytic subunit, GGT – gamma-glutamyltranspeptidase, GPx – glutathione peroxidase, GR – glutathione reductase, GS – glutathione synthetase, GST – glutathione-S-transferase, HSP – heat-shock protein, IAP – inhibitor of apoptosis, Prx – peroxiredoxin, SOD – superoxide dismutase, Sym – *Symbiodinium *spp..

We generated a candidate gene list of groups containing UniSeqs that are likely to be of relevance to cnidarian-dinoflagellate symbioses (Table 3). Among these, the cellular antioxidant-response system could be most comprehensively reconstructed (see below). Genes related to the innate immune system and sugar-binding proteins gave rise to a partial gene inventory (Fig. 1; Table 3). Other genes that are likely to play a role in the cellular events surrounding the breakdown of symbiosis (exocytosis, host-cell detachment, apoptosis and/or autophagy [46-52]) were also identified.

**Table 3:** Potential symbiosis-related genes identified from the *Aiptasia *transcriptome

**Name**	**UniSeq example**	**SwissProt Accession**	**E-value blastx**	**Notes**
***Oxidative stress-/antioxidant activity-related***				
1-Cys peroxiredoxin	CCAS1731.g1	O17433	3.00E-45	Thiol specific antioxidant
Catalase	Contig514	P04040	0	Homolog to catalase
Peroxidase/catalase	Contig1300	O59651	1.00E-26	Catalase and peroxidase activity
Dual oxidase	Contig42	Q9VQH2	8.00E-41	Anti-microbial oxidative burst
L-ascorbate peroxidase T, chloroplastic	CCAS2499.b1	Q42593	2.00E-18	Ascorbate peroxidase activity
Glutathione reductase	CCAS6616.g1	P30635	7.00E-30	Generation of reduced glutathione
Glutamate-cysteine ligase catalytic subunit	CCAS1277.g1	P19468	3.00E-80	
Glutathione synthetase	Contig25	P35668	3.00E-60	Synthesis of glutathione
Glutathione transferase omega-like	Contig320	Q04806	7.00E-21	
Gamma-glutamyltranspeptidase	Contig586	P18956	1.00E-42	Extracellular glutathione breakdown
				
***Apoptosis-/Autophagy-related***				
Apoptosis inhibitor 1	Contig462	Q24306	1.00E-08	
Apoptosis inhibitor 2	CCAS3883.b1	P41454	3.00E-10	
Apoptosis-inducing factor 1	CCAS6130.g1	O95831	9.00E-57	
Apoptosis-inducing factor 2	CCAS2159.g2	Q8BUE4	2.00E-38	
Caspase-3	CCAS2871.b1	Q2PFV2	5.00E-11	Effector caspase
Caspase-8	Contig694	O89110	3.00E-08	Initiator caspase
Caspase-10	CCAS4895.g1	Q92851	3.00E-09	Initiator caspase
Histone-lysine N-methyltransferase HRX	Contig595	Q03164	7.00E-31	Promotes PPP1R15A-induced apoptosis
Death-associated protein kinase 3	Contig472	O88764	9.00E-10	Positive regulator of apoptosis
Large proline-rich protein BAT3	CCAS4314.b1	Q9Z1R2	1.00E-18	Role in ricin-induced apoptosis
Autophagy-related protein 7	CCAS4000.g1	Q641Y5	1.00E-93	
Autophagy-related protein 23	CCAS4265.g1	Q6BHF8	5.00E-06	
Damage-regulated autophagy modulator	CCAS6260.g1	Q5EAK8	1.00E-12	
				
***Endo-, Exo-, Phagocytosis-related***				
Ankyrin-1	CCAS3968.g1	P16157	2.00E-26	Attach membrane proteins to cytoskeleton
AP-1 complex subunit beta-1	CCAS4406.g1	Q10567	5.00E-58	Clathrin-associated adaptor protein complex 1
Dynamin-1	CCAS5621.g1	P21575	7.00E-69	Vesicular trafficking processes
Exocyst complex component 6	Contig181	Q8TAG9	2.00E-21	Component of the exocyst complex
Formin-binding protein 1	CCAS4356.g1	Q96RU3	1.00E-09	Membrane tubulation
Intersectin-1	CCAS6126.g1	Q9Z0R4	4.00E-12	Formation of clathrin-coated vesicles
SNARE-interacting protein KEULE	Contig601	Q9C5X3	5.00E-15	Vesicle trafficking in cytokinesis
Ras-related protein ARA-5	Contig1350	P28188	2.00E-30	Homolog to Rab7
				
***Immunity-related***				
Complement C2	Contig732	Q3SYW2	2.00E-19	
Complement C3	CCAS2220.b1	P23667	5.00E-10	
Complement C5	CCAS2793.b1	P06684	6.00E-11	
Complement component C8 beta chain	CCAS2550.g1	P07358	3.00E-08	
NF-kappa-B inhibitor alpha	CCAS395.g1	P25963	2.00E-21	
				
***Heat-shock-related***				
DnaJ homolog subfamily B member 4	CCAS6247.g1	Q9UDY4	7.00E-45	
DnaJ homolog subfamily C member 10	Contig808	Q9DC23	1.00E-27	
DnaJ homolog subfamily C member 13	Contig104	O75165	7.00E-07	
DnaJ homolog subfamily C member 21	CCAS2838.g1	Q6PGY5	3.00E-56	
DnaJ homolog subfamily C member 7	Contig1270	Q9QYI3	8.00E-42	
Heat shock 70 kDa protein	Contig753	P41753	2.00E-62	
Heat shock 70 kDa protein 1B	Contig573	Q27965	1.00E-170	
Heat shock 70 kDa protein 2	Contig906	P18694	1.00E-12	
Heat shock 70 kDa protein 4	CCAS4411.b1	Q2TFN9	2.00E-22	
Heat shock 70 kDa protein C	Contig926	P19208	6.00E-12	
Heat shock cognate 71 kDa protein	Contig1309	Q71U34	0	
Heat shock factor protein	Contig401	P41154	1.00E-57	
Heat shock protein 81-1	CCAS3866.g1	P27323	9.00E-61	
Heat shock protein 83	CCAS3866.b1	P02828	5.00E-20	
Heat shock protein 90	Contig1047	O44001	6.00E-52	
Heat shock protein homolog SSE1	CCAS4411.g1	Q875V0	7.00E-44	
Heat shock protein HSP 90-alpha	Contig636	O02705	4.00E-29	
Heat shock protein HSP 90-beta	Contig968	Q76LV1	1.00E-180	
				
***Glycan/Lectin-related***				
Calreticulin	CCAS6359.b1	P27798	1.00E-11	Lectin
C-type lectin domain family 4 member F	Contig203	P10716	4.00E-20	Receptor; affinity for galactose/fucose
Ficolin-1	CCAS2736.b1	Q9WTS8	7.00E-13	Binds GlcNAc
Ficolin-2	CCAS3003.b1	Q29041	2.00E-58	Binds GlcNAc
Fucolectin-1	Contig1154	Q9I931	2.00E-13	Binds fucose
Fucolectin-4	CCAS2219.g1	Q9I928	3.00E-12	Binds fucose

Stress-induced photoinhibition and damage to algal photosystem II are thought to be responsible for an increased production of reactive oxygen species [53,54] and consequently, diffusion of hydrogen peroxide (H_2_O_2_) through the membranes into the host cells [55]. The detoxification of H_2_O_2 _requires the activity of catalase or other peroxidases. Superoxide dismutase (SOD), which catalyzes the reduction of superoxide to H_2_O_2_, as well as glutathione peroxidase (GPx), which uses glutathione to detoxify H_2_O_2_, were both not found among the sequenced ESTs. One possibility is that the abundance of SOD transcripts in host cells was low, and the generation of superoxide spatially limited (inside the chloroplasts of *Symbiodinium*). In this case, superoxide may have been efficiently eliminated within the *Symbiodinium *cells, while excess H_2_O_2 _that was not detoxified (e.g., by *Symbiodinium *ascorbate peroxidase), could have diffused into the host cytosol and been reduced to H_2_O and O_2 _by catalase. Alternatively, methodological factors such as insert-size selection or general RNA processing may have prevented the detection of SOD. Other genes that had previously been reported in the context of cnidarian-dinoflagellate symbioses (Additional file 4: Genes that have been studied in the context of cnidarian-dinoflagellate symbiosis, but not found in this study) were also not detected, perhaps for same reasons as discussed above for SOD.

## Conclusion

By analyzing >10,000 high-quality ESTs and generating a comprehensive database for the user community, we have provided a foundation of transcriptomic resources for a symbiotic anemone that is becoming an important model system for studying coral-dinoflagellate symbioses. The set of sequences identified constitutes a rich source of candidate genes that are likely to be involved in processes related to the onset, maintenance, and breakdown of symbiosis. In this context, we were able to reconstruct the oxidative-stress response, which we also found to be prominent during basal transcription. At the current depth of sequencing, we have identified two problems, namely (1) that some transcripts are represented by two (or more) contigs and (2) that we lack information on transcripts of low abundance. These issues will be addressed in the near future by using 454 sequencing, which, for example, has been successfully applied to sequence the coral larval transcriptome of *Acropora millepora *at 3 × coverage [56].

## Methods

### Generation and sequencing of a cDNA library from *Aiptasia pallida* and its dinoflagellate symbiont

A clonal line of *Aiptasia pallida *(clone CC7, available through the Pringle lab) hosting *Symbiodinium *of clade A was established from a single tiny propagule in a population obtained from Carolina Biological Supply (Burlington, NC) and grown into an abundant stock. Given the *Symbiodinium *clade harbored by this population, it is likely that the *Aiptasia *individual originated from the Florida Keys lineage. Approximately 500 anemones of various sizes were harvested from this stock under normal growth conditions (~26°C; salinity, ~33 ppt; light, ~40 μmol m^-2 ^s^-1 ^photosynthetic photon flux; 12-h light-dark cycle), blotted to remove excess water, and immediately frozen in liquid nitrogen. The anemones were then ground to a fine powder under liquid nitrogen using a ceramic mortar and pestle. The powder was weighed (~4 g) while still frozen and mixed with a proportional volume (50 ml) of TRIzol Reagent (Invitrogen, Carlsbad, CA); extraction was then performed in accordance with the manufacturer's instructions yielding ~5 mg of total RNA. This RNA was sent to Open Biosystems (Huntsville, AL), where it was tested for quality; mRNA was then isolated using oligo(dT)-coated magnetic particles (Seradyn, Indianapolis, IN), and cDNA was synthesized. Double-stranded cDNA was size fractionated to enrich for long reads, cloned into the vector pExpress1 (Express Genomics, Frederick, MD), and electroporated into *E. coli *strain DH10B. The resulting library was determined to contain ~96% recombinants with an average insert size of 1.95 kb. Sequencing was performed on 96-well capillary sequencing platforms (ABI 3700) at the DOE Joint Genome Institute (JGI, Walnut Creek, CA) and at the Genome Core Facility at the University of California, Merced, USA, CA.

### Processing of ESTs and implementation of AiptasiaBase

Raw chromatogram files were used as input for the software pipeline EST2uni [36], which was implemented on an Ubuntu server (8.04 "Hardy Heron", Dual Intel Xeon 3.06 GHz) to generate the database named AiptasiaBase [57]. During the pipeline processing, raw EST reads were based-called by phred [37], and quality filtered and vector trimmed by the software Lucy [38]; low-complexity regions and repetitive elements were then removed by seqclean [39] and RepeatMasker [58], respectively. To remove unexpected vector sequences, seqclean additionally screened the processed ESTs using NCBI's UniVec database. All ESTs are available through GenBank accession numbers GH571982 – GH582266.

Clustering of processed ESTs was performed by cap3 [40] with default settings resulting in unique sequences (UniSeqs), for which open reading frames were predicted by ESTScan [59]. Similar UniSeqs were found using BLASTn [60], resulting in clusters of similar UniSeqs [60]. Short-sequence-repeat microsatellites and sequence variations were predicted by Sputnik [61] and local algorithms [36], respectively. All UniSeqs were functionally annotated by BLASTx searches [60] in protein databases nr (GenBank – NCBI), TrEMBL, and SwissProt (Uniprot) [62]; HMMER [63] searches in pfam [42]; and GO-term associations (UniProt GOA, March 2008) [64]. The number of unique genes was estimated by clustering all reverse reads using the cap3 software with default settings.

### BLAST-based prediction of UniSeq origin and KEGG annotation

In order to predict whether an EST originated from *Aiptasia pallida *or *Symbiodinium *spp., we performed a best-BLASTx-hit (BBH) approach (Additional file 2: Flow diagram illustrating BBH approach). First, all UniSeqs were BLASTx-searched (E-value cutoff: 1e-5) in a non-redundant protein database (nr, GenBank, NCBI). If the BBH was from a cnidarian or an alveolate species, the sequence was predicted to originate from *Aiptasia pallida *or *Symbiodinium *spp., respectively, with high confidence. Next, if the BBH was not from a cnidarian or alveolate species, we compared the E-values for the BBHs from a search against nr databases that were previously filtered for sequences from cnidarian (582,480) or alveolate (468,072) species. The organism for which the E-value was lower was assigned to the corresponding UniSeq with medium confidence. Finally, if the E-values for BBH searches in the cnidarian and alveolate databases were equal, we compared the percentage of identical amino acids in the sequence alignments. As in the E-value-based approach, the organism with the higher percentage of identical amino acids was assigned to the corresponding UniSeq (low confidence). In addition to the annotations described above, we used the Automatic Annotation Server provided by the Kyoto Encyclopedia of Genes and Genomes (KEGG) for all UniSeqs using the single-directional best-hit option.

## Authors' contributions

SS carried out the bioinformatics work, analyzed the data, implemented AiptasiaBase, and prepared the manuscript. ECW participated in the data analysis. MT, MLS, and JS participated in the implementation and web-design of AiptasiaBase. CC and JRP cultivated the anemones, and CC prepared the total RNA for cDNA-library construction. JRP, VMW, MM, and JS conceived of the study, coordinated its design, and helped in preparing the manuscript. All authors read and approved the final manuscript.

## Supplementary Material

Additional file 1 **Quality control and assembly statistics**. Click here for file

Additional file 2 **Flow diagram illustrating BBH approach**. Click here for file

Additional file 3 **Detailed EST (N = 10,285) distribution and assignment**. Click here for file

Additional file 4 **Genes that have been studied in the context of cnidarian-dinoflagellate symbiosis, but not found in this study**. Click here for file

## References

[B1] LaJeunesse TC (2002). Diversity and community structure of symbiotic dinoflagellates from Caribbean coral reefs. Marine Biology.

[B2] Rowan R, Powers DA (1992). Ribosomal RNA sequences and the diversity of symbiotic dinoflagellates (zooxanthellae). Proc Natl Acad Sci USA.

[B3] Baird AH, Cumbo VR, Leggat W, Rodriguez-Lanetty M (2007). Fidelity and flexibility in coral symbioses. Mar Ecol Prog Ser.

[B4] Baker AC (2003). Flexibility and specificity in coral-algal symbiosis: Diversity, ecology, and biogeography of *Symbiodinium*. Annu Rev Ecol Evol Syst.

[B5] Little AF, van Oppen MJ, Willis BL (2004). Flexibility in algal endosymbioses shapes growth in reef corals. Science.

[B6] LaJeunesse TC (2002). Diversity and community structure of symbiotic dinoflagellates from Caribbean coral reefs. Mar Biol.

[B7] Santos SR, Shearer TL, Hannes AR, Coffroth MA (2004). Fine-scale diversity and specificity in the most prevalent lineage of symbiotic dinoflagellates (*Symbiodinium*, Dinophyceae) of the Caribbean. Mol Ecol.

[B8] Thornhill DJ, Lajeunesse TC, Kemp DW, Fitt WK, Schmidt GW (2006). Multi-year, seasonal genotypic surveys of coral-algal symbioses reveal prevalent stability or post-bleaching reversion. Mar Biol.

[B9] Wood-Charlson EM, Hollingsworth LL, Krupp DA, Weis VM (2006). Lectin/glycan interactions play a role in recognition in a coral/dinoflagellate symbiosis. Cell Microbiol.

[B10] Rodriguez-Lanetty M, Wood-Charlson EM, Hollingsworth LL, Krupp DA, Weis VM (2006). Temporal and spatial infection dynamics indicate recognition events in the early hours of a dinoflagellate/coral symbiosis. Marine Biology.

[B11] Fitt WK, Trench RK (1983). Endocytosis of the symbiotic dinoflagellate *Symbiodinium microadriaticum *Freudenthal by endodermal cells of the scyphistomae of *Cassiopeia xamachana *and resistance of the algae to host digestion. J Cell Sci.

[B12] Iglesias-Prieto R, Beltran VH, LaJeunesse TC, Reyes-Bonilla H, Thome PE (2004). Different algal symbionts explain the vertical distribution of dominant reef corals in the eastern Pacific. Proc Biol Sci.

[B13] Rowan R, Knowlton N (1995). Intraspecific diversity and ecological zonation in coral-algal symbiosis. Proc Natl Acad Sci USA.

[B14] Rowan R (2004). Coral bleaching: thermal adaptation in reef coral symbionts. Nature.

[B15] Berkelmans R, van Oppen MJ (2006). The role of zooxanthellae in the thermal tolerance of corals: a 'nugget of hope' for coral reefs in an era of climate change. Proc Biol Sci.

[B16] Baker AC (2001). Reef corals bleach to survive change. Nature.

[B17] Buddemeier RW, Fautin DG (1993). Coral Bleaching as an Adaptive Mechanism. BioScience.

[B18] Hoegh-Guldberg O, Jones RJ, Ward S, Loh WK (2002). Communication arising. Is coral bleaching really adaptive?. Nature.

[B19] Hoegh-Guldberg O (1999). Climate change, coral bleaching and the future of the world's coral reefs. Mar Freshw Res.

[B20] Weis VM, Davy SK, Hoegh-Guldberg O, Rodriguez-Lanetty M, Pringle JR (2008). Cell biology in model systems as the key to understanding corals. Trends Ecol Evol.

[B21] Perez SF, Cook CB, Brooks WR (2001). The role of symbiotic dinoflagellates in the temperature-induced bleaching response of the subtropical sea anemone *Aiptasia pallida*. J Exp Mar Biol Ecol.

[B22] Belda-Baillie CA, Baillie BK, Maruyama T (2002). Specificity of a model cnidarian-dinoflagellate symbiosis. Biol Bull.

[B23] Clayton WS (1985). Pedal Laceration by the Anemone *Aiptasia pallida*. Mar Ecol Prog Ser.

[B24] Chen MC, Hong MC, Huang YS, Liu MC, Cheng YM, Fang LS (2005). ApRab11, a cnidarian homologue of the recycling regulatory protein Rab11, is involved in the establishment and maintenance of the *Aiptasia*-*Symbiodinium *endosymbiosis. Biochem Biophys Res Commun.

[B25] Dunn SR, Phillips WS, Green DR, Weis VM (2007). Knockdown of actin and caspase gene expression by RNA interference in the symbiotic anemone *Aiptasia pallida*. Biol Bull.

[B26] Lesser MP (1996). Elevated temperatures and ultraviolet radiation cause oxidative stress and inhibit photosynthesis in symbiotic dinoflagellates. Limnol Oceanogr.

[B27] Nii CM, Muscatine L (1997). Oxidative stress in the symbiotic sea anemone *Aiptasia pulchella *(Carlgren, 1943): Contribution of the animal to superoxide ion production at elevated temperature. Biol Bull.

[B28] Perez S, Weis V (2006). Nitric oxide and cnidarian bleaching: an eviction notice mediates breakdown of a symbiosis. J Exp Biol.

[B29] Sawyer SJ, Muscatine L (2001). Cellular mechanisms underlying temperature-induced bleaching in the tropical sea anemone *Aiptasia pulchella*. J Exp Biol.

[B30] Nagaraj SH, Gasser RB, Ranganathan S (2007). A hitchhiker's guide to expressed sequence tag (EST) analysis. Brief Bioinform.

[B31] Putnam NH, Srivastava M, Hellsten U, Dirks B, Chapman J, Salamov A, Terry A, Shapiro H, Lindquist E, Kapitonov VV (2007). Sea anemone genome reveals ancestral eumetazoan gene repertoire and genomic organization. Science.

[B32] Kortschak RD, Samuel G, Saint R, Miller DJ (2003). EST analysis of the cnidarian *Acropora millepora *reveals extensive gene loss and rapid sequence divergence in the model invertebrates. Curr Biol.

[B33] Leggat W, Hoegh-Guldberg O, Dove S, Yellowlees D (2007). Analysis of an EST library from the dinoflagellate (*Symbiodinium *sp.) symbiont of reef-building corals. J Phycol.

[B34] Schwarz JA, Brokstein PB, Voolstra C, Terry AY, Manohar CF, Szmant AM, Coffroth MA, Miller DJ, Medina M (2008). Coral Life History and Symbiosis: functional genomic resources for two reef building Caribbean corals, *Acropora palmata *and *Montastraea faveolata*. BMC Genomics.

[B35] Kuo J, Chen MC, Lin CH, Fang LS (2004). Comparative gene expression in the symbiotic and aposymbiotic *Aiptasia pulchella *by expressed sequence tag analysis. Biochem Biophys Res Commun.

[B36] Forment J, Gilabert F, Robles A, Conejero V, Nuez F, Blanca JM (2008). EST2uni: an open, parallel tool for automated EST analysis and database creation, with a data mining web interface and microarray expression data integration. BMC Bioinformatics.

[B37] Ewing B, Hillier L, Wendl MC, Green P (1998). Base-calling of automated sequencer traces using phred. I. Accuracy assessment. Genome Res.

[B38] Chou HH, Holmes MH (2001). DNA sequence quality trimming and vector removal. Bioinformatics.

[B39] The Gene Index project. http://compbio.dfci.harvard.edu/tgi/software/.

[B40] Huang X, Madan A (1999). CAP3: A DNA sequence assembly program. Genome Res.

[B41] Consortium TU (2008). The Universal Protein Resource (UniProt). Nucleic Acids Res.

[B42] Finn RD, Tate J, Mistry J, Coggill PC, Sammut SJ, Hotz HR, Ceric G, Forslund K, Eddy SR, Sonnhammer EL (2008). The Pfam protein families database. Nucleic Acids Res.

[B43] Richier S, Rodriguez-Lanetty M, Schnitzler CE, Weis VM (2008). Response of the symbiotic cnidarian *Anthopleura elegantissima *transcriptome to temperature and UV increase. Comp Biochem Physiol D: Genomics Proteomics.

[B44] Matveev IV, Shaposhnikova TG, Podgornaya OI (2007). A novel *Aurelia aurita *protein mesoglein contains DSL and ZP domains. Gene.

[B45] Bogerd J, Babin PJ, Kooiman FP, André M, Ballagny C, Van Marrewijk WJA, Horst DJ Van Der (2000). Molecular characterization and gene expression in the eye of the apolipophorin II/I precursor from *Locusta migratoria*. J Comp Neurol.

[B46] Downs CA, Kramarsky-Winter E, Martinez J, Kushmaro A, Woodley CM, Loya Y, Ostrander GK (2009). Symbiophagy as a cellular mechanism for coral bleaching. Autophagy.

[B47] Dunn SR, Schnitzler CE, Weis VM (2007). Apoptosis and autophagy as mechanisms of dinoflagellate symbiont release during cnidarian bleaching: every which way you lose. Proc Biol Sci.

[B48] Gates RD, Baghdasarian G, Muscatine L (1992). Temperature stress causes host cell detachment in symbiotic cnidarians: implications for coral bleaching. Biol Bull.

[B49] Chen MC, Cheng YM, Sung PJ, Kuo CE, Fang LS (2003). Molecular identification of Rab7 (ApRab7) in *Aiptasia pulchella *and its exclusion from phagosomes harboring zooxanthellae. Biochem Biophys Res Commun.

[B50] Lesser M, Stochaj W, Tapley D, Shick J (1990). Bleaching in coral reef anthozoans: Effects of irradiance, ultraviolet radiation and temperature, on the activities of protective enzymes against active oxygen. Coral Reefs.

[B51] Merle PL, Sabourault C, Richier S, Allemand D, Furla P (2007). Catalase characterization and implication in bleaching of a symbiotic sea anemone. Free Radic Biol Med.

[B52] Sunagawa S, Choi J, Forman HJ, Medina M (2008). Hyperthermic stress-induced increase in the expression of glutamate-cysteine ligase and glutathione levels in the symbiotic sea anemone *Aiptasia pallida*. Comp Biochem Physiol B: Biochem Mol Biol.

[B53] Jones RJ, Hoegh-Guldberg O, Larkum AWD, Schreiber U (1998). Temperature-induced bleaching of corals begins with impairment of the CO_2 _fixation mechanism in zooxanthellae. Plant Cell Environ.

[B54] Warner ME, Fitt WK, Schmidt GW (1999). Damage to photosystem II in symbiotic dinoflagellates: A determinant of coral bleaching. Proc Natl Acad Sci USA.

[B55] Asada K, Takahashi M, Kyle DJ, Osmond CB, Arntzen CJ  (1987). Production and scavenging of active oxygen in photosynthesis. Topics in photosynthesis, Photoinhibition.

[B56] Meyer E, Aglyamova GV, Wang S, Buchanan-Carter J, Abrego D, Colbourne JK, Willis BL, Matz MV (2009). Sequencing and de novo analysis of a coral larval transcriptome using 454 GS-Flx. BMC Genomics.

[B57] AiptasiaBase. http://aiptasia.cs.vassar.edu/AiptasiaBase/index.php.

[B58] RepeatMasker. http://www.repeatmasker.org.

[B59] Iseli C, Jongeneel CV, Bucher P (1999). ESTScan: a program for detecting, evaluating, and reconstructing potential coding regions in EST sequences. Proc Int Conf Intell Syst Mol Biol.

[B60] Altschul SF, Madden TL, Schaffer AA, Zhang J, Zhang Z, Miller W, Lipman DJ (1997). Gapped BLAST and PSI-BLAST: a new generation of protein database search programs. Nucleic Acids Res.

[B61] Sputnik – DNA microsatellite repeat search utility. http://espressosoftware.com/sputnik/index.html.

[B62] TheUniProtConsortium (2008). The universal protein resource (UniProt). Nucleic Acids Res.

[B63] HMMER: biosequence analysis using profile hidden Markov models. http://hmmer.janelia.org.

[B64] Camon E, Magrane M, Barrell D, Binns D, Fleischmann W, Kersey P, Mulder N, Oinn T, Maslen J, Cox A (2003). The Gene Ontology Annotation (GOA) project: implementation of GO in SWISS-PROT, TrEMBL, and InterPro. Genome Res.

