# Supplementary material for: Generation and analysis of transcriptomic resources for a model system on the rise: the sea anemone Aiptasia pallida and its dinoflagellate endosymbiont
Source: BMC Genomics. 2009 Jun 5;10:258. doi: 10.1186/1471-2164-10-258 (PMC2702317; doi:10.1186/1471-2164-10-258)
Supplement: Additional file 1 — Quality control and assembly statistics. [file 1471-2164-10-258-S1.pdf]

Quality control and assembly statistics.

Sequence type	number	discarded by
ESTs	12,896	
Low quality ESTs	2,210	Lucy
short or no insert sequences	210	Lucy
vector sequences	132	Lucy
only poly-A	4	Lucy
short sequences	2	seqclean
short sequences	53	seqclean
High-quality ESTs	10,285	
**Assembly statistics**		
Contigs	1,427	
Singletons	3,498	
UniSeqs	4,925	
Unigenes*	2,564	

* estimated by assembling reverse reads only
