# Supplementary figures and images for: Generation and analysis of transcriptomic resources for a model system on the rise: the sea anemone Aiptasia pallida and its dinoflagellate endosymbiont
Source: BMC Genomics. 2009 Jun 5;10:258. doi: 10.1186/1471-2164-10-258 (PMC2702317; doi:10.1186/1471-2164-10-258)

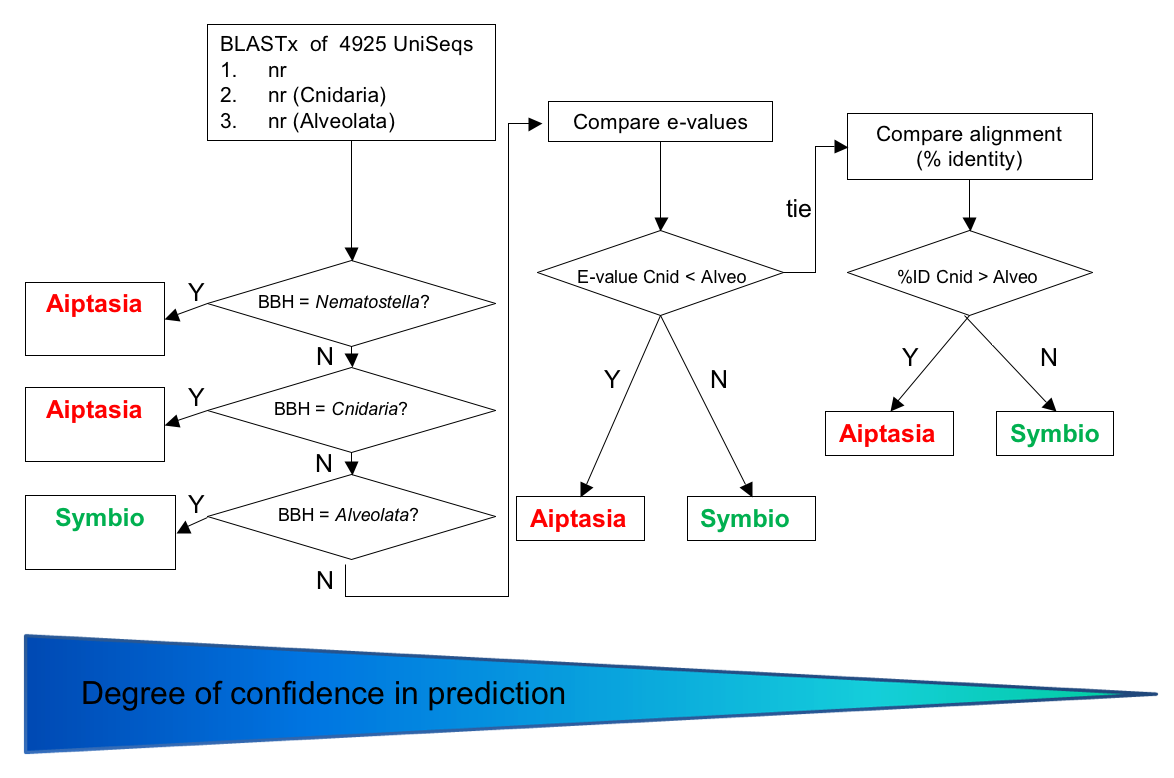

Supplement: Additional file 2 — Flow diagram illustrating BBH approach. [file 1471-2164-10-258-S2.png]
