# Supplementary material for: Generation and analysis of transcriptomic resources for a model system on the rise: the sea anemone Aiptasia pallida and its dinoflagellate endosymbiont
Source: BMC Genomics. 2009 Jun 5;10:258. doi: 10.1186/1471-2164-10-258 (PMC2702317; doi:10.1186/1471-2164-10-258)
Supplement: Additional file 3 — Detailed EST (N = 10,285) distribution and assignment. [file 1471-2164-10-258-S3.pdf]

Detailed EST (N=10,285) distribution and assignment.

Type of UniSeq	#	Best BLASTx hit	If best hit is "other", then evalue	If evalue is equal, then % sim is	Prediction assignment	# of ESTs (%)
Contig	775	Cnidaria			BBH=Cnidaria	4,029 (39.17)
Contig	32	Alveolata			BBH=Alveolata	88 (0.86)
Contig	144	other	is smaller for Cnidaria		evalue(Cnidaria) < evalue(Alveolata)	540 (5.25)
Contig	63	other	is smaller for Alveolata		evalue(Alveolata) < evalue(Cnidaria)	228 (2.22)
Contig	14	other	equal	higher for Cnidaria	sim%(Cnidaria) > sim%(Alveolata)	576 (5.60)
Contig	7	other	equal	higher for Alveolata	sim%(Alveolata) > sim%(Cnidaria)	27 (0.26)
Contig	2	other	equal	equal	no assignment	5 (0.05)
Contig	390	no hit			no hit	1,294 (12.58)
Singlet with sister read predicted as BBH=Cnidaria	330				BBH=Cnidaria	330 (3.21)
Singlet with sister read predicted as BBH=Alveolata	2				BBH=Alveolata	2 (0.02)
Singlet with sister read predicted as evalue(Cnidaria) < evalue(Alveolata)	17				evalue(Cnidaria) < evalue(Alveolata)	17 (0.17)
Singlet with sister read predicted as evalue(Alveolata) < evalue(Cnidaria)	14				evalue(Alveolata) < evalue(Cnidaria)	14 (0.14)
Singlet with sister read predicted as sim%(Cnidaria) > sim%(Alveolata)	8				sim%(Cnidaria)>sim%(Alveolata)	8 (0.08)
Singlet that hit Cnidaria or Alveolata	1,401	Cnidaria			BBH=Cnidaria	1,401 (13.62)
Singlet that hit Cnidaria or Alveolata	89	Alveolata			BBH=Alveolata	89 (0.87)
Singlet that hit other with sister read in Contig that has no hit	36	other	is smaller for Cnidaria		evalue(Cnidaria) < evalue(Alveolata)	36 (0.35)
Singlet that hit other with sister read in Contig that has no hit	30	other	is smaller for Alveolata		evalue(Alveolata) < evalue(Cnidaria)	30 (0.29)
Singlet that hit other with sister read in Contig that has no hit	4	other		higher for Cnidaria	sim%(Cnidaria) > sim%(Alveolata)	4 (0.04)
Singlet that hit other with sister read in Contig that has no hit	4	other		higher for Alveolata	sim%(Alveolata) > sim%(Cnidaria)	4 (0.04)
Singlet that hit other and have no sister reads in Contigs	254	other	is smaller for Cnidaria		evalue(Cnidaria) < evalue(Alveolata)	254 (2.47)
Singlet that hit other and have no sister reads in Contigs	131	other	is smaller for Alveolata		evalue(Alveolata) < evalue(Cnidaria)	131 (1.27)
Singlet that hit other and have no sister reads in Contigs	45	other		higher for Cnidaria	sim%(Alveolata) > sim%(Cnidaria)	45 (0.44)
Singlet that hit other and have no sister reads in Contigs	30	other		higher for Alveolata	sim%(Cnidaria) > sim%(Alveolata)	30 (0.29)
Singlet with no hit (sister read is in Contig)	571	no hit			no hit	571 (5.55)
Singlet with no hit (neither sister read is in Contig)	532	no hit			no hit	532 (5.17)
