# Supplementary material for: Generation and analysis of transcriptomic resources for a model system on the rise: the sea anemone Aiptasia pallida and its dinoflagellate endosymbiont
Source: BMC Genomics. 2009 Jun 5;10:258. doi: 10.1186/1471-2164-10-258 (PMC2702317; doi:10.1186/1471-2164-10-258)
Supplement: Additional file 4 — Genes that have been studied in the context of cnidarian-dinoflagellate symbiosis, but not found in this study. [file 1471-2164-10-258-S4.pdf]

Genes that were studied in the context of cnidarian-dinoflagellate symbiosis, but not found in this study.

Gene	Reference
Aiptasia pallida cyclophilin mRNA, complete cds	Perez and Weis 2008
Aiptasia pallida caspase-like protein mRNA, complete cds	Dunn *et al.* 2006
Aiptasia pallida abhp mRNA, complete cds	Dunn *et al.* 2006
Anthopleura elegantissima copper/zinc superoxide dismutase-like protein mRNA, complete cds.	Rodriguez-Lanetty *et al.* 2006
Anthopleura elegantissima prohibitin protein-like protein mRNA, partial cds	Rodriguez-Lanetty *et al.* 2006
Anthopleura elegantissima sphingosine 1-phosphate phosphatase 2-like protein-like mRNA, complete sequence	Rodriguez-Lanetty *et al.* 2006
Anemonia viridis copper/zinc superoxide dismutase CuZnSODa mRNA, complete cds	Plantivaux *et al.* 2004
Anemonia viridis copper/zinc superoxide dismutase CuZnSODb mRNA, complete cds	Plantivaux *et al.* 2004
